# Leadless Pacing: Current Status and Ongoing Developments

**DOI:** 10.3390/mi16010089

**Published:** 2025-01-14

**Authors:** Richard G. Trohman

**Affiliations:** Section of Electrophysiology, Division of Cardiology, Department of Internal Medicine, Rush University Medical Center, 1653 W. Congress, Chicago, IL 60612, USA; rtrohman@rush.edu; Tel.: +1-(312)-942-5000

**Keywords:** leadless pacing, lead limitations, leadless advantages/disadvantages

## Abstract

Although significant strides have been made in cardiac pacing, the field is still evolving. While transvenous permanent pacing is highly effective in the management of bradyarrhythmias, it is not risk free and may result in significant morbidity and, rarely, mortality. Transvenous leads are often the weakest link in a pacing system. They may dislodge, fracture, or suffer breaches in their insulation. This review was undertaken to clarify leadless risks, benefits, and alternatives to transvenous cardiac pacing for bradyarrhythmias and heart failure management. In order to clarify the role(s) of leadless pacing, this narrative review was undertaken by searching MEDLINE to identify peer-reviewed clinical trials, randomized controlled trials, meta-analyses, and review articles, as well as other clinically relevant reports and studies. The search was limited to English-language reports published between 1932 and 2024. Leadless pacing was searched using the terms Micra™, Nanostim™, AVEIR™, single-chamber leadless pacemaker, dual-chamber leadless pacemaker, cardiac resynchronization therapy (CRT), cardiac physiological pacing (CPP) and biventricular pacing (BiV). Google and Google Scholar, as well as bibliographies of identified articles were also reviewed for additional references. The advantages and limitations of leadless pacing as well as options that are under investigation are discussed in detail.

## 1. Introduction

The rich history of cardiac pacing began more than 90 years ago. Its evolution has resulted from a combination of creative thought and dramatic advances in technology. Table 1 summarizes key developments in transvenous cardiac pacing (additional details are available in Supplement S1) [1,2,3,4,5,6,7,8,9,10,11,12,13,14,15,16,17,18,19]. This manuscript aims to provide a review of the structure, function, and limitations of transvenous cardiac pacemaker leads, the development of leadless pacemakers, the structural characteristics of leadless pacemakers, clinical trials that investigated the safety and efficacy of leadless pacing, implantation techniques, indications for leadless pacing, risks and complications of leadless pacing, advantages and disadvantages of leadless pacing, investigational devices (those without U.S. Food and Drug Administration [FDA] approval, without CE Mark or without both), and final conclusions.

## 2. Structure, Function, and Limitations of Transvenous Cardiac Pacemaker Leads

**Figure 1:**
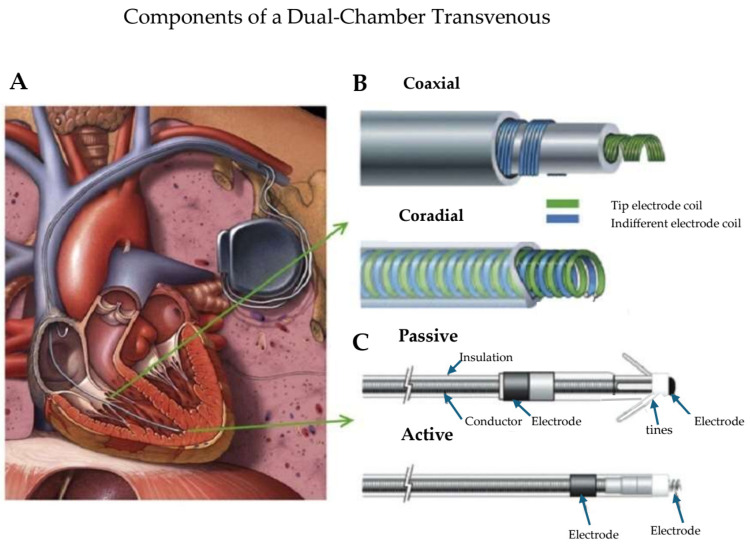
(**A**) Transvenous pacemaker systems primarily consisting of a hermetically encased can (also known as a generator) containing the battery and its circuitry placed subcutaneously (in the pre-pectoral region) or submuscularly between the pectoral muscles. The can is connected to the myocardial tissue by a pacemaker lead (or leads). The leads contain conductor coils to the distal electrodes separated by insulation material. (**B**) The leads are of coaxial (coil within a coil) or coradial (side-by-side coils) design depending on the arrangement of the conductor coils. (**C**) The lead tips are attached to the myocardium by a screw-like penetrating helix (active fixation) or by tines that embed in the myocardial trabeculations (passive fixation). Adapted from reference [20] with permission.

Transvenous pacemaker systems consist primarily of a hermitically sealed can, placed in the pre-pectoral region, containing the battery and all circuitry. The can is connected to the myocardial tissue by a pacemaker lead or leads. The leads contain conductor coils to the distal electrodes separated by insulation material [20] (Figure 1). Over the last three to four decades, the basic material used for most conductors has been MP-35N (SPS Technologies, Cleveland, OH, USA), an alloy of nickel, cobalt, chromium, and molybdenum. High electrical resistance has been overcome with the development of composite-wire conductors that incorporate low-resistance metals such as silver and stainless steel, with high-strength materials such as titanium, platinum, and platinum–iridium alloy. The leads are of coaxial (coil within a coil) or coradial (side-by-side coils) design, depending on the arrangement of the conductor coils. Lead tips are attached to the myocardium by a penetrating helix (active fixation) or by tines that embed in the myocardial trabeculations (passive fixation) [20]. Standardization of pacemaker leads has allowed global compatibility across manufacturers. The international standard-1 (IS-1) terminal ring and pin arrangement allows (with some exceptions) connection to pacemaker generators from different manufacturers [21].

Despite advances made in cardiac pacing, lead-related issues remain the “Achilles heel” of cardiac pacing. Apart from infection, there is a greater incidence of lead-related complications compared with issues related to pulse generators.

Early lead-related issues include dislodgement resulting in loss of capture, as well as under-sensing due to an acute inflammatory response. The latter is usually remediable via reprogramming.

Lead insulation provides physical and electrical shielding of the conductor elements. In addition, insulation contributes significantly to the structural strength of the entire lead body [22]. Among the problems with the major materials used for lead insulation (polyurethane, silicone rubber, fluoropolymers) is that they were not originally specifically designed for this purpose. Each has disadvantages when used as part of a biological pacing system. Because leads are subject to repetitive mechanical stress during each cardiac cycle and by shoulder girdle movement in the body, leads are the most common pacemaker components to fail. Insulation breaks result in low impedance measurements and oversensing of signals generated by surrounding muscle structures because the conductors are exposed [22].

Over the last three to four decades, the basic material used for most conductors has been MP-35N (SPS Technologies, Cleveland), an alloy of nickel, cobalt, chromium, and molybdenum. The main advantage of MP-35N is high strength and resistance to corrosion [23]. Its main disadvantage is its high electrical resistance. This has been overcome with the development of composite-wire conductors that incorporate low-resistance metals such as silver and stainless steel with high-strength materials such as titanium, platinum, and platinum–iridium alloy [23]. Conductor fracture typically results in non-physiologic signals (“noise”) caused by the lead. This noise consists of high-frequency, saturated electrograms generated by intermittent contact between disrupted conductor elements (called filars) and can be associated with elevated lead impedance and loss of capture. Chronic inflammation (at times due to an underlying primary cardiomyopathic process) may result in loss of capture. Acute venous entry angles, medial venous access near the costoclavicular ligament, sharp turns in the pocket, young age, subpectoral device placement, tight sutures, and silicone insulation are risk factors associated with insulation breaks and lead fractures [20]. Other lead problems include infection/endocarditis, venous thrombosis and emboli, and tricuspid regurgitation [20].

In addition to the problems noted above, leads may be misplaced in the left chamber of the heart. Although inadvertent malpositioning of cardiac implantable electronic device leads into the left ventricle is an uncommon complication of transvenous pacing and defibrillation, it may result in serious consequences. In 2016, Ohlow et al. reported a 3.4% incidence of inadvertent lead placement into the left heart; however, this included the cardiac veins [18]. Inadvertent endocardial left ventricular (LV) lead placement creates a nidus for thrombus formation and possible embolization. Treatment of LV lead misplacement discovered late after implantation includes lead removal or chronic anticoagulation with warfarin to prevent thromboemboli. Although LV lead extraction was first described in 1991 [19], procedural safety remains uncertain. Because use of dabigatran in patients with mechanical heart valves was associated with increased rates of thromboembolic and bleeding complications compared with warfarin, substituting a direct oral anticoagulant for warfarin in the setting of malpositioned left ventricular leads is not recommended [24].

Rapid identification of lead position is critical during implantation and just after the procedure, with immediate correction required if malpositioning is detected. If lead misplacement is discovered late after implantation, the lead should be surgically removed or chronic anticoagulation with warfarin should be initiated [24].

## 3. Development of Leadless Pacemakers (LPs)

Leadless cardiac pacing has been developed in response to lead-related problems and the desire to reduce the incidence of device-related infection. Because leads have long been considered the weakest link of cardiac pacing systems, a totally self-contained cardiac pacing system was conceptualized more than 50 years ago. In 1970, Spickler and associates reported totally self-contained leadless cardiac pacing in a canine model. Nevertheless, this concept has become a reality only recently as a result of technological advancements in battery energy, endocardial fixation and delivery systems [25]. The first report of leadless pacing in humans was published in 2014 [26].

## 4. Structure of Leadless Pacemakers

The newest versions of leadless pacemakers available from Medtronic (Minneapolis, MN, USA) are Micra AV and Micra VR2. Each has a length of 25.9 mm, an outer diameter of 6.7 mm, and a mass of 1.75 g. Materials in chronic contact with human tissue include titanium, titanium nitride, parylene C, PEEK, nitinol, platinum–iridium alloy, and silicone rubber. Nitonal FlexFix^TM^ tines are used for fixation. A monolithic controlled release device (MCRD) provides steroid elution to help maintain acceptable pacing thresholds. The nominal pacing cathode measures 2.5 mm^2^, is point-sintered and coated with titanium nitride. The minimum pacing anode measures 22 mm^2^ and is coated with titanium nitride. The cathode-to-anode spacing is 18 mm. Both have a 3.2-volt lithium-hybrid CFx silver vanadium oxide battery [27,28].

The AVEIR leadless ventricular pacemaker has a length of 38 mm, a diameter of 6.5 mm. and a mass of 2.4 g. The outer shell/can of the device is composed of titanium. Fixation is achieved via a nonretractable helix. Its tip electrode is a titanium nitride-coated, platinum–iridium disk located at the center of the fixation helix, which measures about 2.2 mm^2^. The tip electrode includes a single dose of dexamethasone sodium phosphate (DSP) intended to reduce inflammation. The ring electrode is the uncoated part of the titanium pacemaker case, and its surface area measures > 127 mm^2^. The inter-electrode space is >24 mm [29].

The AVEIR leadless atrial pacemaker has a length of 32.2 mm, a diameter of 6.5 mm. and a mass of 2.1 g. The LP distal tip electrode comprises a titanium nitride-coated, platinum–iridium helix (LSP201A) or disk (LSP202V) located at the center of the fixation helix. The tip electrode includes a single dose of dexamethasone sodium phosphate (DSP) intended to reduce inflammation. The ring electrode is the uncoated part of the titanium pacemaker case, and its surface area measures ~124 mm^2^. The inter-electrode space is >24 mm [29]. All leadless conductors use MP-35N alloys. Table 2 provides additional comprehensive details of leadless pacing devices [30].

## 5. Clinical Trials Investigating Leadless Pacing’s Safety and Efficacy

LEADLESS was a prospective, non-randomized, single-arm multicenter study of the safety and clinical performance of a completely self-contained leadless cardiac pacemaker (Nanostim Inc., Sunnyvale, CA, USA). The 33 patients enrolled had a mean age of 77 ± 8 years, and 67% of the patients were male. Implantation was successful in 97% (32/33) of the cohort. Five patients (15%) required the use of more than one pacing device due to inadvertent LV placement, malfunction of the release knob, delivery catheter damage, damage to the device’s helix, and difficulty with the delivery catheter’s wire deflection mechanism. The one inadvertent device placement in the LV (via a patent foramen ovale) was successfully retrieved without sequelae. A new device was implanted in the RV apex. Another patient developed cardiac tamponade. He underwent emergent median sternotomy on cardiopulmonary bypass and surgical repair of an RV apical perforation. Despite gradual recovery, he suffered left-sided hemiplegia attributable to a right-sided main cerebral artery ischemic infarct and died on post-procedure day 18 [26].

Follow-up of the other 31 patients who underwent successful implantation revealed no pacemaker-related adverse events reported between 3 and 12 months of follow-up. At 6 and 12 months of follow-up, the pacing performance results were as follows: mean pacing threshold (at a 0.4 ms pulse width), 0.40 ± 0.26 Volts [V] and 0.43 ± 0.30 V; R-wave amplitude 10.6 ± 2.6 millivolts [mV] and 10.3 ± 2.2 mV; and impedance 625 ± 205 Ohms [Ω] and 627 ± 209 Ω. At the 12-month follow-up, 61% of the patients had their rate response sensor activated. Adequate rate response was observed in all of these patients [26,31].

A subsequent report from the Leadless II study [32] reviewed data from the first 300 Nanostim (acquired by St. Jude Medical, Sylmar, CA, USA) device recipients followed for 6 months as well as the entire cohort of 526 patients enrolled as of June 2015. The primary composite efficacy end point was an acceptable pacing capture threshold (≤2.0 V at 0.4 ms) and an acceptable sensing amplitude (R wave ≥ 5.0 mV, or a value equal to or greater than the value at implantation) through 6 months.

Implantation was successful in 289 of the initial 300 patients (96.3%) and 270 patients (90%) had an acceptable primary composite end point. Inadequate pacing capture thresholds were noted in 4 patients. Inadequate sensing was noted in 14 patients and one patient had inadequate pacing and sensing parameters [32].

The primary safety end point was freedom from serious device-related adverse events during the initial 6 months post-implantation. Twenty-two serious device-related adverse events occurred in 20 patients (6.7%). Included among these complications were cardiac perforation (4 [1.3%]), device dislodgement (5 [1.7%], elevated pacing thresholds requiring device replacement (4 [1.3%]) and vascular complications in 4 [1.3%] patients [32].

There were 28 deaths (5.3%) in the total cohort, 19 (68%) occurred within 6 months, 8 (29%) between 6 and 12 months, and 1 (3%) after 12 months. The mean age of patients who died was 79.1±10.9 years. Two deaths (0.4%) were classified by the clinical events committee as procedure-related [32].

St. Jude Medical (now Abbott) halted implantation of its Nanostim leadless pacemaker in October 2016, due to reports of battery malfunction resulting in loss of telemetry and pacing output. St. Jude Medical had previously halted implantations of the Nanostim leadless pacemaker after reports surfaced of problems with the device’s docking button, which was designed to connect with the retrieval catheter, allowing the Nanostim device to be retrieved and removed after implantation.

The redesigned version of Nanostim, Aveir LP (Abbott Cardiovascular, Plymouth, MN, USA) incorporated important design improvements, including the use of standard transvenous pacemaker lithium carbon-monofluoride battery chemistry with a 12% (1.1 years) longer battery life (up to 10.4 years), an altered form factor (10% shorter, 1.5-F wider, to 19.5-F), a modified docking button (facilitating retrievability), a modified delivery system with an ergonomic design, and a new application-specific integrated circuit (ASIC) chip designed to support a dual-chamber pacing system once approved [33].

The LEADLESS II–Phase 2 trial evaluated the efficacy and safety of the AVEIR LP system in 200 patients with standard VVI(R) pacing indications. The primary efficacy end point was a composite score of acceptable pacing thresholds (≤2.0 V at 0.4 ms) and R-wave amplitudes (≥5.0 mV) at implantation through six weeks of follow-up.

The implant success rate was 98% and 83.2% did not require repositioning. The primary safety end point “serious complications” occurred in eight patients. The most common complications were cardiac tamponade and premature device deployment. The primary efficacy end point was achieved in 188 of 196 (95.9%) patients who underwent successful device implantation [33].

**Figure 2:**
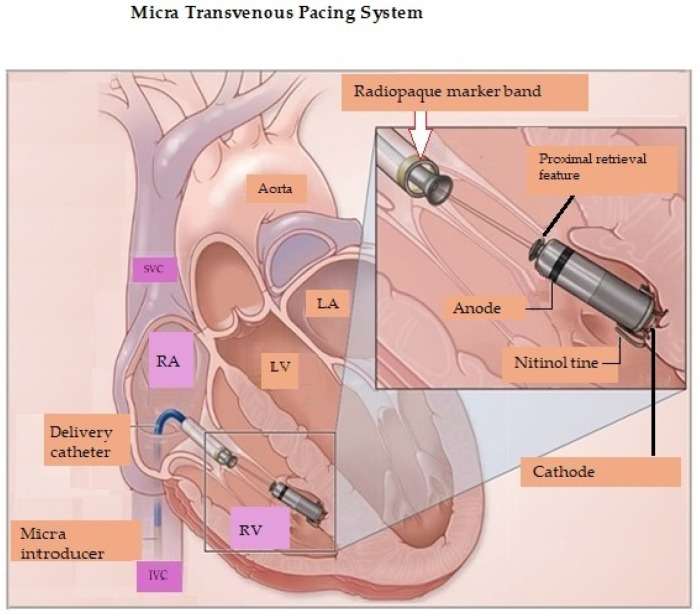
Micra transvenous pacing system positioned in the right ventricle. RA = Right atrium; RV = Right ventricle; LA = Left atrium; LV = Left ventricle; SVC = Superior vena cava; IVC = Inferior vena cava. Reproduced from reference [35] with permission.

Shortly before the time when implantation of Nanostim was halted, Ritter and colleagues reported early experience with implantation of the Micra transcatheter [leadless] pacing system (TPS, Model MC1VR01, Medtronic plc, Mounds View, MN, USA) [34]. Micra (physical characteristics noted above) was a single-chamber ventricular pacemaker (Figure 2) [34]. A total of 140 patients underwent device implantation. The prespecified safety goal was >85% freedom from unanticipated serious adverse device-related events and efficacy was assessed via the mean 3-month pacing capture threshold. During a mean follow-up of 1.9 ± 1.8 months, there were no unanticipated serious adverse events. However, 30 adverse events related to the system or procedure occurred (primarily transient arrhythmias or femoral access complications) [34]. Among patients followed for 3 months (*n* = 60), the mean pacing threshold was 0.51 ± 0.22 V, and none exceeded 2 V. The mean R-wave amplitude was 16.1 ± 5.2 mV, and the mean impedance was 650.7 ± 130 Ω [34].

In 2016, Reynolds et al. reported an interim (6-month) analysis of the safety and efficacy of the Micra transcatheter pacing system. The device was successfully implanted in 719 of 725 patients (99.2%). The primary efficacy end point was the percentage of patients with low (≤2 V at a pulse width of 0.24 ms) and stable pacing thresholds (an increase of ≤1.5 V from the time of implantation) at 6 months post-implantation. The primary safety end point was freedom from system-related or procedure-related major complications [35].

The primary efficacy goal was evaluated in 297 patients and was achieved in 292 (98.3%). In comparison to a historical (transvenous pacing) cohort, the safety profile was comparable to that of a transvenous system while providing low and stable pacing thresholds [35].

There were 28 major complications in 25 patients (4%), including four of six patients who underwent unsuccessful attempts at implantation. Among these, one death occurred, eleven patients had cardiac perforation or pericardial effusions, two patients had venous thrombosis (one also had a pulmonary embolus), two had elevated pacing thresholds and five developed arteriovenous fistulae at their femoral venous entry site [35].

For Micra™, the long term (12 months) safety objective of freedom from major complications was achieved in 96%. Four new major complications occurred. Three patients developed heart failure and one was associated with pacemaker syndrome (atrioventricular dyssynchrony associated with a constellation of symptoms such as dyspnea, fatigue, and exercise intolerance) [36,37]. Although there were 26 patients with 33 systemic infectious events during the trial, none were attributed to implantation of the device.

Among the 630 patients with pacing threshold data available at 12 months, 93% had a threshold of ≤1 V (mean 0.60 ± 0.38 V) at 0.24 ms pulse duration, and out of the 58 patients with available pacing threshold data at 24 months, 97% had a pacing threshold of ≤1 V (mean 0.53 ± 0.23 V) also at 0.24 ms. Pacing thresholds tended to decrease after implant and subsequently remained stable [36]. R-waves (ventricular sensing) were 15.1 mV at 12 months and 15.5 mV at 24 months [36].

In 2017, the acute performance of the Micra transcatheter pacemaker was reported from a worldwide post-approval registry. Performance of the Micra transcatheter pacemaker in the real-world setting demonstrated a high rate (99.6%) of implant success and low rate (1.51%) of major complications over 30 days post-implant. The rates of pericardial effusion, device dislodgement, and infection were low, reinforcing the results of the investigational study [38].

The Longitudinal Coverage With Evidence Development Study on Micra Leadless Pacemakers (Micra CED) is a continuously enrolling observational cohort study evaluating complications, utilization, and outcomes of leadless VVI pacemakers in the US Medicare fee-for-service population. At a 5-year follow-up, data from the post-approval registry on 1809 patients enrolled between July 2015 and March 2018 revealed that Micra leadless pacemaker outcomes continued to demonstrate low rates of major complications and system revisions as well as an extremely low incidence of infection. There were no Micra removals due to infection. At 36 months, system revision rates were significantly lower with Micra compared to transvenous systems (3.2% vs. 6.6%, *p* < 0.001) [39].

**Figure 3:**
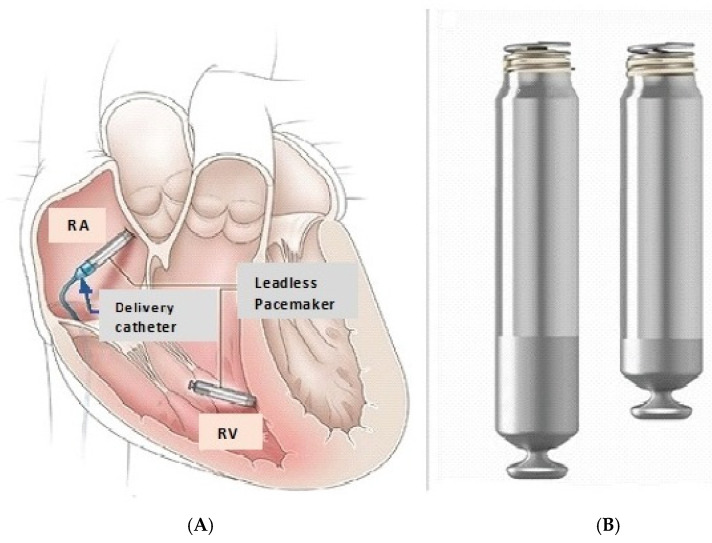
(**A**) Device placement. The RV LP is positioned at the interventricular septum aiming to reduce the risk of perforation. (**B**) The RA LP measures 5.8 mm and is shorter than the RV LP. It is ideally positioned at the ostium of the RA appendage. RA = Right atrium; RV = Right ventricle. Adapted from reference [40] with permission.

Because single-chamber ventricular pacemakers do not provide atrial pacing or consistent atrioventricular synchrony, implantation is limited to approximately 20% of patients who have indications for a pacemaker [40,41]. In 2023, Knops and colleagues reported 90-day results from 300 patients who received a dual-chamber leadless pacing system AVEIR DR (Abbott Cardiovascular, Plymouth, MN, USA) [40]. The system consisted of two devices implanted percutaneously (in a single procedure), one in the right atrium and one in the right ventricle. AVEIR AR LP was designed to accommodate the right atrial size and sensitivity, with unique features designed to achieve implant stability and optimization. A 1.63 mm inactive outer helix provide primary fixation while the recessed inner helix acts as the pacing electrode while also providing additional fixation and electrical stability [30,40]. As previously noted, the atrial leadless pacemaker is shorter (32.2 mm in length). The right ventricular leadless pacemaker is physically identical to the commercially available single-chamber leadless device. Both leadless devices are 6.5 mm in diameter (Figure 3). The leadless pacemakers wirelessly communicated bidirectionally (implant-to-implant), on a beat-to-beat basis via a series of short pulses delivered through the blood and myocardial tissue after each locally paced or sensed event, thus maintaining atrioventricular synchrony [40].

Procedural success was attained in 295 of 300 patients (98.3%). The atrial pacing device was not implanted in 2 patients, and 3 had inadequate implant-to-implant communication. Intraprocedural device dislodgment (6; 5 atrial) was successfully managed with retrieval and repositioning. Five additional dislodgements occurred at 26 ± 17 days post procedure. Another atrial device was implanted in three patients. The authors recommended targeting the ostium of the appendage to optimize implant-to-implant communication and possibly limit atrial lead dislodgement [40]. Eight revision procedures were performed. The indications for these revisions were atrial dislodgement (6), suboptimal implant-to-implant communication (1), and intermittent ventricular capture (1). Successful percutaneous retrieval was achieved in each instance. Six new leadless pacemakers were implanted successfully. At the discretion of the investigator, two patients did not receive a replacement atrial leadless pacemaker [40].

Four patients died. The deaths were adjudicated (by an independent clinical events committee) to be unrelated to the device or the procedure [40].

The first primary performance end point, a combination of adequate atrial capture threshold (≤3.0 V at 0.4 ms) and atrial sensing amplitude (P wave of ≥1.0 mV) at the 3-month visit was achieved in 90.2%. The second primary performance end point was AV synchrony at the 3-month visit, defined as a paced or sensed ventricular beat within 300 ms of a paced or sensed atrial beat in ≥70% of the cardiac cycles evaluated during a 5 min seated recording, and was found in 97.3% [40]. The FDA-approved the AVEIR™ dual chamber (DR) leadless pacemaker system on 5 July 2023 [42,43].

**Figure 4:**
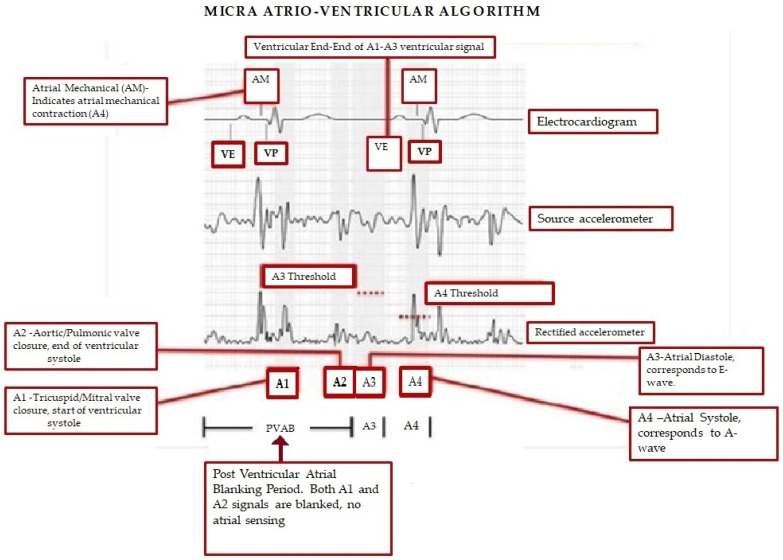
Micra AV accelerometer signals and their relationship to surface ECG wave. AM, atrial mechanical; AV, atrioventricular; ECG, electrocardiography; PVAB, post-ventricular atrial blanking; VE, end of ventricular ectopy. Reproduced from reference [44] with permission.

Unlike traditional dual-chamber transvenous permanent pacemakers, which sense atrial electrical activity directly through a lead implanted in the right atrium, the Micra AV algorithm (Medtronic, Inc., Minneapolis, MN, USA) identifies mechanical atrial contraction, detected by the device implanted in the ventricle, and allows AV synchronous pacing. The algorithm relies on a three-axis accelerometer to detect atrial contraction [44]. Micra AV accelerometer signals and their relationship to surface ECG waves are depicted in Figure 4. The A1 signal corresponds to closure of the tricuspid and mitral valves and the onset of ventricular isovolumic contraction. Hence, A1 falls at the end of the electrocardiographic (ECG) QRS complex (electrical systole precedes mechanical systole). The A2 signal corresponds to aortic and pulmonic valve closures, corresponding to the end of ventricular systole. Hence, the A2 signal typically falls at the end of T wave. The A3 signal corresponds to passive ventricular filling while the A4 signal corresponds to atrial contraction. These (A3 and A4) signals correspond to the E and A mitral inflow echocardiographic measurements [44]. In Micra AV2, the AV conduction mode switch to a lower rate is programmable to facilitate AV conduction. A3 and A4 thresholds were improved. The Auto+ A3 adjusts the A3 threshold more appropriately to facilitate tracking at higher sinus rates. The auto A4 threshold could be adjusted too high in Micra AV. Therefore, a programmable max A4 threshold was added in Micra AV2 with a nominal value of 3.0 m/s^2^ (meters per second squared). A change in battery composition and a decrease in current drain increased longevity by 4+ years for Micra AV2 and Micra VR2 devices. Micra AV2 and Micra VR2 have expanded labeling for MRI scans < 1.5T [45].

The MARVEL 2 (Micra Atrial tRacking using a Ventricular accELerometer 2, Minneapolis, MN, USA) study assessed the ability to provide AV synchronous pacing by mechanically sensing atrial contractions via a right ventricular Micra leadless pacemaker [46]. The algorithm facilitated AV synchrony ≥ 70% at rest in 95% of patients with complete atrioventricular block (AVB) [46]. Micra AV2 was approved by the FDA in 2020 based on the results of the MARVEL 2 study [45].

The AccelAV study was a prospective, non-randomized, multicenter clinical trial conducted in the United States and Hong Kong and reported in 2023 [47]. The primary aim of the AccelAV study was to characterize chronic atrioventricular synchrony in patients implanted with Micra AV (Model MC1AVR1, Medtronic, Inc., Minneapolis, MN, USA). As noted above, Micra AV is implanted in the RV and uses the device’s accelerometer to mechanically sense atrial contractions and facilitate VDD pacing. In this mode, V = ventricular pacing, D = sensing in the atrium and ventricle, D = an intrinsic QRS can inhibit ventricular pacing, and an intrinsic P-wave can trigger an AV delay resulting in P-wave tracking and maintenance of AV synchrony via RV pacing [48]. In complete AV block, the intrinsic P-wave does not conduct to the ventricle and the end of the AV delay is followed by a paced ventricular complex [48].

Among 54 patients with normal sinus node function and complete AVB, Micra AV mean resting AV synchrony was 85.4% at 1 month, and ambulatory AV synchrony was 74.8%. In a subset of 20 patients with programming optimization, mean ambulatory AV synchrony was 82.6% [47]. Medtronic received the CE mark for these devices in January 2024 [49].

## 6. Implantation Techniques

The vast majority of leadless pacemakers have been implanted via the following sequence: Right femoral vein access, introduction and advancement of a large (e.g., 27 F) sheath under fluoroscopy navigating the delivery system to superior vena cava and the right interventricular septum, deployment, and fixation. If difficulty with venous stenosis, occlusion, or severe tortuosity of the right femoral vein is encountered, a left femoral venous approach may also be used [50]. As previously noted, right atrial devices are ideally advanced to the ostium of the right atrial appendage [40].

In addition to the difficulties noted above, femoral vein access may be accompanied by complications in the groin area, such as hematomas, arteriovenous fistulae, or arterial pseudoaneurysms, each occurring with an approximate risk of 1%. Moreover, stenosis or a tortuous anatomy of the inferior vena cava (IVC) may hinder the successful implantation of a femoral leadless pacemaker. Therefore, Molitor et al. compared peri-procedural safety and efficacy in the first 100 consecutive patients who underwent Micra™ leadless pacemaker implantation via the right internal jugular vein (at two centers) to the first 100 patients using a femoral implantation approach at the University Hospital Zurich. The mean procedure (35.63 ± 10.29 versus 48.9 ± 21.0 min; *p* < 0.01) and fluoroscopy times (4.66 ± 5.16 min versus 7.7 ± 7.8 min; *p* < 0.01) were shorter compared to the femoral approach. Electrical parameters were similar between the two techniques. Two complications occurred during jugular veinous implantation (1 pericardial effusion and 1 dislocation), versus 16 complications using the femoral approach (1 pericardial effusion, 2 femoral artery injuries, and 13 major groin hematomas). This difference was statistically significant (*p*= 0.0005) [51]. Additional experience with this technique is needed to shed further light on its efficacy and safety.

El-Chami and Shah have suggested ways to avoid ventricular perforation (see Section 8, below). They suggest: (1) Always advancing the delivery system over a stiff wire under fluoroscopic guidance; (2) Withdrawing the delivery sheath in the right atrium rather than advancing the delivery system out of the sheath; (3) Avoiding traumatic manipulation of the delivery system by starting counter-clockwise rotation in the lower one-third of the RA to steer the delivery system anteriorly toward the tricuspid valve; (4) Avoiding suddenly popping the delivery system across the tricuspid valve and into the right ventricle; (5) Making sure the delivery system is free in the right ventricle and applying clockwise torque toward the right ventricular septum (to avoid the risk of perforation [see below] in the inferior right ventricular recess and apex); and (6) Avoiding frequent deployments (>5) if a good position or electrical characteristics are not achieved [50].

## 7. Indications for Leadless Pacing

Transvenous permanent pacing is typically performed by accessing the subclavian and axillary veins via puncture, or the cephalic vein via cutdown to implant transvenous leads [52]. Common acute transvenous system-related issues include lead dislodgement, thoracic trauma, vascular injury, pocket hematoma, and infection. Common long-term transvenous pacemaker complications (which may require transvenous lead extraction) include lead conductor fractures, abnormal lead sensing or pacing values, insulation failures, device header or connector problems, premature battery depletion, and pocket infection [53]. Leadless permanent pacing was developed to bypass the two major weaknesses of the transvenous systems, the lead(s) and the subcutaneous (or submuscular) pocket [54,55,56]. They are an excellent alternative approach in case of specific comorbidities, such as limited upper venous access, transvenous pacemaker infection (see below) and kidney failure patients receiving hemodialysis (who are also likely to have limited central venous access) [54,56]. While femoral implantation of transvenous leads is possible, it is technically challenging and is a far less desirable option [57].

The initial indication for leadless pacing therapy was mainly limited to patients who had persistent or permanent AF with a slow ventricular response. Single chamber ventricular leadless pacing may also be indicated in patients with paroxysmal atrioventricular (AV) block, sinus node disease or syncope, in which infrequent ventricular pacing is expected [54]. A European Heart Rhythm Association (EHRA) survey regarding the use of leadless pacing in Europe revealed that, among 52 centers from 21 countries, the most common indications for leadless pacing were permanent AF (83%), a history of complications with a conventional pacemaker (87%), anticipated difficult vascular access (91%) and an expected higher risk of infection (70%) [54,58].

The 2021 European Society of Cardiology (ESC) guidelines on cardiac pacing and cardiac resynchronization therapy provided similar recommendations for leadless pacing. As noted above, the guidelines recommended patients without upper-extremity venous access (making the usual transvenous pacing approaches impossible or, at least, impractical) as the most appropriate candidates (ESC Class IIa-B). The low infection rates associated with leadless pacing were noted to make LP attractive for patients on hemodialysis and those with a previous history of a pacemaker infection (ESC Class IIa-B). These guidelines also recommended that LP be considered in all single-lead pacemaker candidates, such as those with permanent atrial fibrillation and patients likely to have a low pacing burden (ESC Class IIb-C). Given the uncertainty surrounding the optimal LP replacement strategy, older patients (with a limited life expectancy) may be more suitable candidates for leadless pacing [30,59].

In the previously noted prospective, multicenter, single-group study to evaluate the safety and performance of a dual-chamber leadless pacemaker system, the most common indications for dual-chamber pacemaker implantation were sinus-node dysfunction (190 patients [63.3%]) and atrioventricular block (100 patients [33.3%]) [40].

## 8. Risks and Complications of Leadless Pacing

Despite the potential advantages of leadless pacing (see below), the risk of procedural complications is far from insignificant. In 2022, Haddadin et al. reported the rate of complications in 7821 patients who underwent leadless pacemaker implantation (Table 3) [60]. Immediate procedure-related complications occurred in 7.5% of patients. Pericardial effusion that did not require pericardiocentesis occurred in 1.9% of patients, and pericardiocentesis was performed in 1.0%. Vascular complications occurred in 2.3% of patients (0.33% required repair), and device dislodgment occurred in 0.51%. The most significant predictors of procedural complications were end-stage renal disease (odds ratio [OR] 1.65; 95% confidence interval [CI] 1.17–2.32; *p* = 0.004), congestive heart failure (OR 1.28; 95% CI 1.01–1.62; *p* = 0.04), and coagulopathy (OR 1.77; 95% CI 1.34–2.34; *p* < 0.001). All-cause readmission occurred in 17.9% of patients within 30 days of device implantation, and 1.36% were procedure-related. At 30 days post-implant, 0.25% of patients needed a new pacemaker, and 0.18% had pericardial complications [60]. It is important to understand that these data were extracted from the United States National Readmission Database (NRD), between 2016 and 2018. Therefore, it is likely that this study reflects a relatively early clinical experience. A substantial operator learning curve exists for LP implantations, and the implanters might not have been at the end of their learning phase. Additional safety data from large (>500 patients) single-chamber LP large registries and regulatory trials is summarized in Table 4 [30]. Table 5 compares complication rates between patients implanted with leadless versus transvenous pacemakers

## 9. Advantages and Disadvantages of LPs

Table 6 [61,62,63,64,65,66,67,68,69,70,71,72,73] contrasts the advantages and disadvantages of leadless pacing. One of the major drawbacks of LPs is the higher rate of perforation and pericardial effusion at implantation compared to transvenous pacing systems. LP-related perforations are not only more common, but also more severe [30]. LP-related perforations are associated with a high number of deaths, tamponades, and rescue thoracotomies. A 2021 analysis from the MAUDE database revealed that 96% of reported major adverse events with LPs were related to perforation and 27% of major adverse events required a sternotomy [69]. Patient characteristics associated with an increased risk of cardiac perforation included advanced age, female sex, low body mass index (<20), chronic obstructive pulmonary disease, heart failure, prior myocardial infarction, COPD, absence of prior cardiothoracic surgery, and dialysis [30,70]. A recent position paper recommended that LP implantation should preferably be performed at centers with on-site cardiothoracic surgery support [30,71]. For RV pacing, placement of the device at the interventricular septum rather than the apex may reduce the likelihood of perforation (see Figure 2 and Figure 3).

Despite their potential problems, LPs are associated with a substantially lower overall complication rate, mainly caused by a low rate of long-term complications [30]. The major advantage of LPs is the elimination of lead and pocket-related complications. Avoiding pocket and lead revision reduces the rate of infection. This predominantly reflects the lack of a subcutaneous pocket (the major source of device-related infection) and to a lesser extent the absence of transvenous leads [30]. In a sub-analysis of Micra PAR, 105 patients had Micra LP implanted after extraction of an infected TV-PPM (37% of Micra were implanted the same day as device removal and lead extraction) [65,69]. No reinfection of the Micra LP was seen [65].

## 10. Investigational Devices *

* = Without FDA approval, without CE mark or without both.

Boston Scientific has investigated the use of a Modular CRM (modular cardiac rhythm) management system (mCRM™). The system combines use of a subcutaneous ICD (EMBLEM™ S-ICD) and a leadless pacemaker EMPOWER™ LP. Modular CRM therapy aims to reduce the risk of transvenous leads while providing the option to pace for bradycardia or receive antitachycardia pacing (ATP) for ventricular tachyarrhythmias [66].

Knops et al. recently reported results from a multinational, single-group study that enrolled patients at risk of sudden death from ventricular arrhythmias and followed them for six months after implantation of the modular pacemaker–defibrillator system [67]. The investigators enrolled 293 patients and 151 completed the 6-month follow-up period. Wireless device communication was successful in 98.8% of communication tests and 97.5% were free from leadless pacemaker-related major complications. Pacing thresholds ≤ 2.0 V were achieved in 97.4% [67].

The combined system exceeded performance goals for freedom from major complications. Eight patients died. None of the deaths were judged to be related to arrhythmias or the implantation procedure [67]. Boston Scientific will pursue FDA approval for the EMPOWER leadless pacemaker and mCRM system in 2025 [73].

The subcutaneous ICD (S-ICD) was developed to avoid the vascular risks of transvenous ICDs. The first S-ICD, the SQRX (Cameron Health, San Clemente, CA, USA), was approved by the United States Food and Drug Administration in 2012. Cameron Health was acquired by Boston Scientific (Natick, MA, USA) and the second and third generations of the device were released in 2015 and 2016 [74]. In a secondary analysis of the PRAETORIAN trial, significantly fewer lead-related complications and systemic infections occurred in the S-ICD group compared with the TV-ICD group (*p* < 0.001, *p* = 0.03, respectively). In addition, more complications required invasive interventions in the TV-ICD group compared with the S-ICD group (8.3% vs. 4.3%, HR: 0.59; *p* = 0.047) [75]. Medtronic has developed an extravascular implantable cardioverter-defibrillator (ICD) that has a substernally implanted single lead to enable pause-prevention pacing, antitachycardia pacing, and defibrillation energy, similar to transvenous ICDs [76]. While none of these devices are leadless, they send a clear message that transvenous leads are an imperfect option.

**Figure 5:**
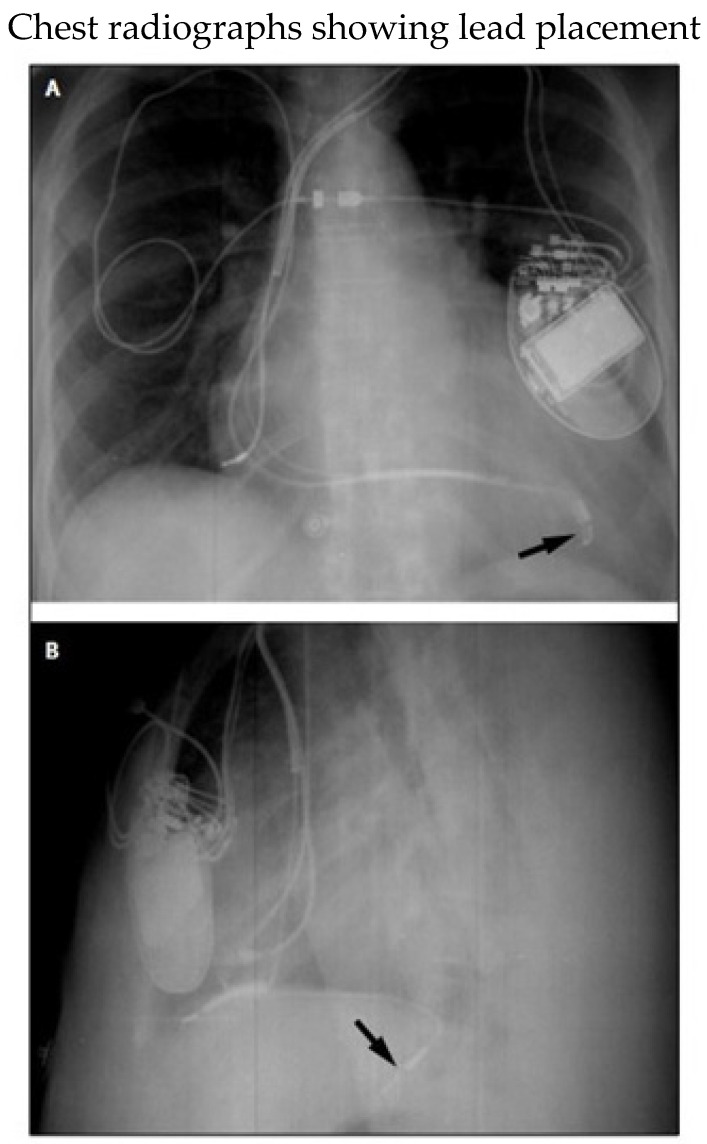
(**A**) Posteroanterior and (**B**) lateral radiographs showing tip of left ventricular lead (arrows) in a tributary of the middle cardiac vein. Despite proximity to the left hemidiaphragm, phrenic near stimulation did not take place. The right ventricular lead points anteriorly toward the rib cage. Reproduced from reference [78] with permission.

Cardiac physiological pacing (CPP) refers to any form of cardiac pacing intended to restore or preserve synchrony of ventricular contraction. Biventricular (BiV) pacing is the most common method used to achieve resynchronization. Left ventricular leads are usually implanted epicardially via the coronary sinus (CS) into the cardiac veins (Figure 5), ideally targeting areas of late activation (most often the lateral or posterolateral wall) [77,78]. When CRT cannot be obtained with a CS LV lead due to anatomical or functional considerations, options include surgical placement of an epicardial lead, His-bundle pacing and left bundle branch pacing [77]. His-bundle pacing has been limited by relatively high pacing thresholds and lead instability. Left bundle branch pacing has been increasingly used because it overcomes those issues and is more likely to result in the narrowing of the QRS complex when conduction disease is more distal [79].

Reasons for failure or abandonment of CRT with BiV pacing include: venous inaccessibility (subclavian, innominate vein, or superior vena cava occlusion), CS inaccessibility (occlusion, dissection, perforation, obstructive Thebesian valve), cardiac vein inaccessibility (small, angulated, or tortuous vein branches), suboptimal vein location (non-lateral vein, anterior interventricular vein), persistent SVC, poor lead stability (prone to dislodgment), high-capture thresholds, diaphragmatic stimulation, and major complications such as pericardial effusion/tamponade, CS or vascular dissection, sustained ventricular tachyarrhythmias/cardiac arrest, pulmonary embolism, respiratory failure or stroke [77]. These issues make potential leadless options exciting.

**Figure 6:**
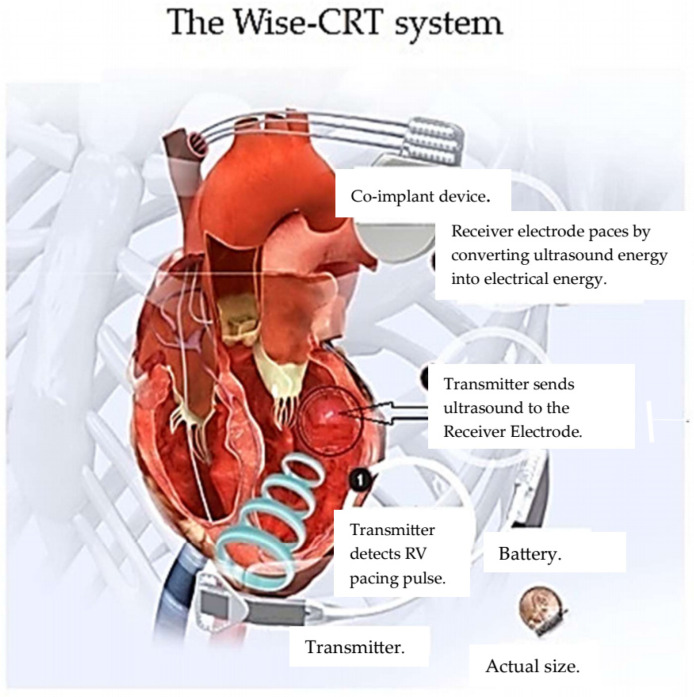
Adapted from reference [81] with permission.

The Wireless Stimulation Endocardial for Cardiac Resynchronization (WiSE-CRT) system (EBR Systems, Sunnyvale, CA, USA) includes a receiver electrode (9.1 mm  ×  2.7 mm) that can be implanted (via a retrograde aortic or transseptal approach) in the left ventricular endocardium, a transmitter implanted in the intercostal space that detects RV pacing and delivers ultrasound energy to the receiver electrode, and a battery. The receiver electrode transforms ultrasound energy into electrical energy which results in LV pacing [69,80] (Figure 6).

In the SELECT-LV (Safety and Performance of Electrodes implanted in the Left Ventricle) multi-center non-randomized trial, 35 patients received the new WiSE-CRT system after failing conventional CRT. Implantation was successful in 34 patients (97.1%). Biventricular pacing was seen in 33 patients (97.1%) at one month. Clinical improvement was achieved in 84.8% of patients. An improvement > 5% in left ventricular ejection fraction was noted in 66%. Serious complications within 24 h occurred in 3 patients. Additional complications occurred in 8 patients after 24 h. These included VF during implantation in one patient, electrode embolization, groin complications, pocket complications, and one cerebrovascular accident [69,81].

Subsequently, 90 patients from 14 European centers underwent WiSE-CRT system implantation. The system was successfully implanted in 85 (94.4%) patients. Improvement in heart failure symptoms occurred in seventy percent of patients. However, acute (<24 h), 1- to 30-day, and 1- to 6-month complications rates were 4.4%, 18.8%, and 6.7%, respectively. Five deaths (5.6%) occurred within 6 months (three were procedure-related). Because 76% of complications occurred within centers’ first 10 procedures, the authors speculated that a learning curve was likely involved in implantation outcomes [69,82].

Data presented at a late-breaking session at Heart Rhythm Society 2023 from the SOLVE-CRT study revealed that the safety and efficacy end points were met with a 16.4% improvement in cardiac function (*p* = 0.003) and an absence of device and procedure-related complications in 80.9 percent of patients (*p* < 0.001) [83,84,85]. The FDA granted the WiSE-CRT system a Breakthrough Device designation, supporting priority review and paving the way for premarket approval [85]. The WiSE-CRT system received European CE mark approval in 2015.

In 2019, Funasako and colleagues reported two cases of a totally leadless biventricular pacing approach. The Micra Transcatheter Pacemaker System was used for RV pacing and was combined with the WiSE-CRT wireless endocardial pacing system to achieve their goal [86].

The first patient had long-standing persistent atrial fibrillation (AF) with a rapid ventricular response. Following Micra implantation, AV junction ablation was performed. Unfortunately, the patient developed LV systolic dysfunction (EF 33%) and heart failure. Subsequently, the WiSE-CRT system was added. At a 6-month follow-up, the patient reported symptomatic improvement and transthoracic echocardiography revealed normalization of the LV ejection fraction [86].

The second patient had previously undergone mitral and tricuspid annuloplasty along with a bilateral maze procedure for persistent AF. However, AF continued, and the patient suffered episodes of complete AV block. A Micra device was implanted 1-year post-cardiac surgery. Due to progressive LV dysfunction (EF 25%), a WiSE LV system was implanted [86].

One week after a WiSE LV system was implanted, the patient’s symptoms of heart failure resolved. At a 6-month follow-up, the patient was completely asymptomatic and reported large increases in exercise tolerance and quality of life [86].

In 2021, Carabelli et al. reported eight patients with indications for both Micra and WiSE-CRT systems due to one of the following: (a) heart failure related to a high burden of RV pacing by Micra; (b) the need to remove a previously infected CRT system and/or perceived persistent high risk of further system infection; or (c) anatomical conditions such as venous obstruction or difficult coronary sinus anatomy that resulted in failed attempts at conventional CRT system implantation [69,87].

Similarly to the procedure described in Funasako’s report [86], the WiSE system was implanted in two steps. The battery was implanted subcutaneously at the midaxillary line and connected to the transmitter. The transmitter was placed in the fourth to sixth intercostal spaces lateral to the left parasternal border at a site (confirmed by echocardiography) with a lung- and bone-free acoustic window to the left ventricle [87]. Subsequently, a combination of fluoroscopy, echocardiography, electrical timing and pacing thresholds was used to identify an appropriate endocardial LV pacing site. Once this was determined, the electrode was deployed and anchored into the LV endocardium [87].

Seven patients reached the 6-month follow-up (one died at 4 months due to acute heart failure). The others had significant improvement in left ventricular EF (+11.29 ± 8.46%; *p* = 0.018) and four patients had an improvement in LVEF ≥10% [87]. In both studies, QRS duration decreased after the WiSE system was turned on [86,87].

## 11. Conclusions

Leadless pacing is an exciting clinical option and is associated with rapidly evolving technology. It offers physicians and many patients a viable alternative to transvenous pacing. It is particularly enticing as a way to reduce procedural morbidity, particularly the risk of infection, because pocket formation is not needed. It offers an opportunity to replace an infected transvenous device and minimize or eliminate recurrent infection. It is clear that implanters require a learning curve, and lack of experience may result in more complications. Cardiac perforation and pericardial effusions/cardiac tamponade remain the most problematic issues. Although likely to be controversial, recommendations suggest that leadless device implantation should not take place in centers that do not perform cardiac surgery. Optimal management of these devices at end of life remains uncertain and time will tell whether extraction or simply adding a new device is more advantageous.

## Figures and Tables

**Table 1:** Evolving concepts in cardiac pacing [1,2,3,4,5,6,7,8,9,10,11,12,13,14,15,16].

Date (Year)	Investigator (s)	Milestone
1932	Hyman	Used a machine that produced electricity and plunged a needle through the chest wall into the heart for resuscitation of cardiac standstill.
1950	Bigelow et al.	Introduced a bipolar lead via the right internal jugular vein and stimulated the right atrium during open-heart surgery.
1952	Zoll	Developed external pacing.
1958	Furman	Used a transvenous electrode to successfully stimulate the right ventricle (RV) for 96 days.
1958	Lillihei and Bakken	Reported efficacy of a battery-powered external pacemaker in 18 patients.
1958	Senning and Elmqvist	Implanted the first pacemaker using an epicardial lead.
Circa 1965	Berkovitz	Credited with the innovation of demand (signal-sensed) pacing. Introduced the concept of Universal DDD pacing.
1977	Funke	Introduced atrial synchronous and atrioventricular (AV) sequential (DDD) pacing.
1981	Rickards and Norman	Designed a physiologically adaptive cardiac pacemaker which sensed the interval between the delivered stimulus and the evoked T wave and used the stimulus-evoked T wave interval to set the subsequent pacemaker escape interval.
1994	Cazeau et al.	Contributed a landmark report of successful four-chamber [biventricular (BiV)] pacing for heart failure. Initially, left ventricular (LV) lead placement was surgical.
1998	Daubert et al.	Described permanent left ventricular pacing via leads advanced to the coronary sinus and positioned in the cardiac veins.
1999	Auricchio et al.	Used balloon occlusive angiography (for road map of cardiac veins), reshaped guide catheters, and advanced leads over guidewires.
2018	Arnold et al.	His bundle pacing provided better ventricular resynchronization and improvement in hemodynamics compared to biventricular pacing.
2020	Ponnusamy et al.	Left bundle branch pacing (LBBP) effective in overcoming His-bundle pacing’s limitations, providing lead stability, low stable pacing thresholds, and correcting distal conduction system disease.
2022	Vijayaraman et al.	Conduction system pacing improved clinical outcomes compared to biventricular pacing in a large cohort of patients with an indication for cardiac resynchronization therapy.

**Table 2:** Characteristics of leadless pacemakers.

	Nanostim	Aveir VR	Micra VR ^a^	Micra AV ^b^	Aveir AR ^c^	Empower ^d^
Dimensions (mm)	42 × 5.99	38 × 6.5	25.9 × 6.7	25.9 × 6.7	32.2 × 6.5	32.0 × 6.1
Volume (cc)	1.0	1.1	0.8	0.8	1.0	0.75
Sheath size, F, ID/OD	18/21	25/27	23/27	23/27	25/27	21/23
Pacing mode	VVI(R)	VVI(R)	VVI(R)	VVI(R) or VDD(R)	AAI(R)	VVI(R) + SCD diraected ATP
Able to use as dual chamber LP	No	Yes	No	No	Yes	No
Fixation	Screw-in helix	Screw-in helix	4 nitinol tines	4 nitinol tines	Screw-in helix	4 nitinol tines
Battery	Lithium carbon monofluoride	Lithium carbon monofluoride	Lithium-hybrid carbon monofluoride silver vanadium oxide	Lithium-hybrid carbon monofluoride silver vanadium oxide	Lithium carbon monofluoride	Lithium carbon monofluoride
Battery longevity at standard settings (years)	N/A	9.9 VVIR 7.3 DDDR	4.7 VVIR	4.8 VVIR	6.8 AAI(R) 5 DDI(R)	N/A
Battery longevity at alternate setting ^e^	N/A	16.1 (1.25 V at 0.4 ms. 60 bpm, 100% VP, 500 ohm, single chamber mode); 9.8 (1.25 V at 0.4 ms. 60 bpm, 100% VP, 500 ohm, dual chamber mode).	9.6 (1.5 V at 0.4 ms. 60 bpm, 100% VP, 500 ohm)	8.6 (1.5 V at 0.4 ms. 60 bpm, 100% VP, 500 ohm)		N/A
MRI-compatible	1.5 T	1.5 T, 3 T	1.5 T, 3 T	1.5 T, 3 T	1.5 T, 3 T	1.5 T, 3 T
Remote monitoring	No	No	Carelink	Carelink	No	No
Magnet mode	100 bpm for 8 cycles, then rate dependent on battery status	100 bpm for 5 cycles, then rate dependent on battery status	No	No	Yes, AOO (VOO in case of dual chamber pacing) at 100 bpm for 5 cycles, then rate dependent on battery status	N/A

^a^ Micra VR2: similar values but estimated battery longevity 6.3 years (at ISO standard programming: 2.5 V at 0.24 ms, 60 beats/min, 100% VP, 600 ohm, VVIR mode) or 12.4 years (1.5 V at 0.24 ms, 60 beats/min, 100% VP, 600 ohm, VVIR/VVI mode). ^b^ Micra AV2: similar values but estimated battery longevity 6.1 years (at ISO standard (at ISO standard programming: 2.5 V at 0.24 ms, 60 beats/min, 100% VP, 600 ohm) or 11.6 years (1.5 V at 0.24 ms, 60 beats/min, 100% VP, 600 ohm, VDD mode). ^c^ Atrial LP can be connected to the Aveir VR LP to provide dual chamber leadless pacing. Regulatory approval for sole atrial use has been granted. ^d^ Currently does not have regulatory approval. ^e^ Battery longevity is shown for the ISO standard and an alternative setting more applicable to real world use. Adapted from reference [30], with permission.

**Table 3:** Leadless pacemaker implant complications. In-hospital clinical and procedural outcomes.

**Clinical Outcomes**	
All-cause death	513 (6.6)
Acute venous thromboembolism	443 (5.7)
Acute stroke	285 (3.6)
Any bleeding	1179 (15.1)
Blood transfusion	693 (8.9)
**Immediate procedural outcomes**	
Total procedure-related complication rates *	588 (7.5)
All vascular complications	181 (2.31)
Vascular complications requiring repair	26 (0.33)
Procedure-related bleeding	194 (2.48)
Pericardial effusion without requiring pericardiocentesis	146 (1.9)
Pericardial effusion without pericardiocentesis	82 (1.0)
Thoracotomy among patients with effusion ^†^	26 (11.5)
Device dislodgment	40 (0.51)
Removal or repositioning of leadless pacemaker	253 (3.25)
**Resource Utilization**	
Post-procedure length of stay (d)	2 days (1–6)
Cost (US$)	$34,483 (23,602–57,040)

Values are given as n (%) or median (IQR). IQR = interquartile range. * Total procedure-related complications included vascular complications, pericardial effusion, device dislodgment, and procedure-related bleeding. ^†^ The percentage of thoracotomy was calculated among patients with pericardial effusion (n = 228). Reproduced from reference [60] with permission.

**Table 4:** Short-term and long-term complications of leadless pacing.

	Leadless	Leadless II	Leadless Observational	Leadless II Phase 2	Micra IDE	Micra PAR	MAP EMEA	Italian Registry	Total
LP model	Nanostim	Nanostim	Nanostim	Aveir VR	Micra VR	Micra VR	Micra VR	Micra VR	
Short-term complication rate, %	6.1	5.8	5.3	4.8	2.9	2.5	2.6	0.5	3.0
No. of patients	33	718	300	210	726	1809	928	665	5389
Follow-up duration, months	3	1	6 ^a^	1.5	1	1	1	1	1.3
Pericardial effusion/cardiac perforation	3.0	1.5	1.3	1.9	1.4	0.4	0.6	0.0	0.8
Dislodgement during procedure	0.0	0.3	0.0	1.4	0.0	0.1	0.0	n/a	0.1
Dislodgement after procedure	0.0	1	0.3	0.0	0.0	0.1	0.0	0.2	0.2
Vascular complications	0.0	2.2	1.3	1.0	1.2	0.6	1.1	0.2	0.7
Other	3.0	1.1	3.0	1.0	0.7	1.6	0.9	0.2	1.4
Long-term complication rate, % ^b^	3.0	0.6	n/a	1.9	1.1 ^c^	1.8	1.0	0.0	1.1
No. of patients	33	718	n/a	210	726	1809	928	665	5089
Mean follow-up duration, months	38 ^d^	10.6 ^d^	n/a	14.4	16.4	51.1 ^d^	9.7	39 ^e^	29.7
Dislodgement	0.0	0.0	n/a	0.0	0.0	0.0	0.0	0.0	0.0
Infection	0.0	0.0	n/a	0.0	0.0	0.1	0.1	0.0	0.0
Other	3.0	0.6	n/a	1.9	1.1	1.8	1.1	0.0	1.2

Event rates are unadjusted rates in patients with ≥1 occurrence(s) of the specified complication (except for “Other” which is the combined number or rate of “Other” complications). ^a^ All complications occurred within 3 months. ^b^ Occurring after short-term follow-up period. ^c^ Combined 30 days to 6 months and >6 months rates. ^d^ Median. ^e^ Median of LP and TV-PM cohort combined. Adapted from reference [30] with permission.

**Table 5:** Complications between cohorts patients implanted with a leadless pacemaker compared to patients implanted with a transvenous pacemaker.

			All Complications					
			LPs		TVPMs			
	Type of Analysis	Follow-Up (Months)	n	Rate, %	n	Rate, %	*p*-Value	HR (95% CI)
Leadless II (Nanostim)	Matched.1:2 short-term complications. Matched. 1:2 long-term complications.	1 LPs 10.6 TV-PM 13.4 ^b^	718 718	5.8 0.6	1435 1435	9.4 4.9	0.010 <0.001	Overall 0.44 (0.32–0.60)
Micra IDE (MicraVR)	Unmatched, unadjusted ^a^ Matched. 1:1.	12 12	726 726	4.0 4.0	2667 726	7.6 n/a	0.001 <0.001	0.46 (0.35–0.77) 0.52 (0.30–0.72)
Micra PAR (MicraVR)	Unmatched, unadjusted. Adjusted rates of complications.	36-month estimate 36-month estimate	1809 1809	4.1 n/a	2667 2667	8.5 n/a	<0.001 <0.001	0.47 (0.36–0.61) 0.43 (0.29–0.65)
Micra CED (MicraVR)	Adjusted rates of short-term complications. Adjusted rates of long-term complications. ^c^	1 3-year estimate	5746 6219	7.7 4.9	9662 10,212	7.4 7.1	0.49 <0.0001	Not reported 0.68 (0.59–0.78)
Micra AV CED (Micra AV)	Adjusted rates of short-term complications. Adjusted rates of long-term complications. ^c^	1 6-month estimate	7471 7471	8.6 3.5	107,800 107,800	11.0 7.0	<0.0001 <0.0001	Not reported 0.50 (0.43–0.57)
U.S. data (NRD 2017-2019; Micra VR) ^d^	Unmatched, unadjusted.	In hospital	5986	16.0	131,746	6.4	<0.001	Not reported
U.S. data (NIS 2017-2019; Micra VR) ^d^	Unmatched, unadjusted. Matched. 1:1.	In hospital In hospital	16,825 3084	8.6 8.0	565,845 3084	11.2 13.2	<0.001 <0.001	Not reported Not reported
Italian Registry (MicraVR ^d^)	Unmatched, unadjusted. Matched. 1:1.	39 ^e^ 39 ^e^	665 442	0.5 0.7	2004 442	2.8 1.3	0.003 0.129	Not reported Not reported

Event rates are the rates of patients with ≥1 complication. ^a^ Occurring after the short-term window. ^b^ Median. ^c^ includes all complications after implantation. ^d^ Studies may overlap. ^e^ Median of LP and TVPM combined. Adapted from reference [30] with permission.

**Table 6:** Strengths and weaknesses of leadless pacing.

LP Advantages	LP Disadvantages
Reduced risk of pocket infection, hematoma LP implantation after extraction of an infected TVPM has been performed without recurrent infection [65]	Potential cardiac perforation, effusion, and tamponade [69,70,71,72]
Should be strongly considered in dialysis recipients to preserve upper extremity venous access and limit the risk of transient bacteremia and device infection [64]	Inexperienced operators may have poorer results [30,68]
No risk of lead dislodgement, fracture or insulation break	Device may dislodge and retrieval may be needed (this is not always easy or even feasible)
AVEIR DR uses 2 devices to provide AV synchrony [62] Micra AV permits atrial tracking and ventricular pacing	Micra limited to single chamber pacing
Cosmetic: No chest incision or bulging [61]	Uncertain whether old or dysfunctional devices should be routinely removed
Micra VR2 provides rate response	No defibrillation capabilities * [61,65,66]
Battery life of single chamber devices comparable to transvenous devices (~16–17 years) [49]	Battery life of dual chamber devices reduced (particularly atrial device in AVEIR DR [6.4 years]) [62]
Usually safe for MRI, but there may be a limit based on the strength of the magnet in the MRI machine [61]	Indications are evolving and are incompletely defined.
Length of hospital stay may be shorter [67]	30-day all-cause readmission rates have been reported to be significantly higher (17.9% vs. ~13%) than for transvenous PPM procedures [60,63]

* Solutions in development.

## Supplementary Materials

The following supporting information can be downloaded at: https://www.mdpi.com/article/10.3390/mi16010089/s1. The supplement is referenced independently of the main text.
