# Supplementary material for: Leadless Pacing: Current Status and Ongoing Developments
Source: Micromachines (Basel). 2025 Jan 14;16(1):89. doi: 10.3390/mi16010089 (PMC11767621; doi:10.3390/mi16010089)
Supplement: Supplementary file 1 [file micromachines-16-00089-s001.zip › micromachines-3152823-supplementary.pdf]

Supplement S1: A Brief History of Transvenous pacing

In 1932, Alfred Hyman reported the use of a machine that produced electricity and was introduced into the heart by a needle plunged through the chest wall for resuscitation of cardiac standstill[1,2,3]. The concept was not accepted by the medical community and received criticism for interfering with natural events[2].

In 1950, Bigelow and associates introduced a bipolar lead via the right internal jugular vein and stimulated the right atrium during open-heart surgery[2,3,4]. In 1952. Zoll developed external pacing[2,3,5].
Three 1958 developments paved the way for modern cardiac pacing[2,3]. Furman used a transvenous electrode to successfully stimulate the right ventricle (RV) for 96 days. Lillehei and Bakken reported efficacy of a battery-powered external pacemaker in 18 patients. Later that year, Senning and Elmqvist implanted the first pacemaker using an epicardial lead[2,3].

Berkovits has been credited with the innovation of demand (signal-sensed inhibition) pacing[2,3,6] and introduced the concept, that he called the Universal pacemaker (atrial synchronous and atrioventricular [AV] sequential pacing). In 1977 Funcke introduced atrial synchronous and atrioventricular sequential (DDD) pacing. Effective rate responsive pacing became available in the early 1980s[3,7].

A landmark report of successful four chamber [biventricular (BiV)] pacing for HF was published by Cazeau and colleagues in 1994[3,8]. Initial left ventricular (LV) lead placement was surgical. Daubert and associates introduced a transvenous approach in 1998[3,9]. Aided by interventional cardiology cross training, Auricchio and colleagues recognized important obstacles to percutaneous LV pacing. They realized that preshaped catheters would help access anatomical variations in coronary sinus branches and that balloon occlusive angiography would create roadmaps of cardiac venous anatomy. Additionally, they understood that novel leads designed to facilitate successful placement (easily advanced over guidewires) were essential[10,11]. This approach became CRT's mainstream method [3,10,11]. Overcoming these impediments was pivotal in making CRT a standard of HF care.

Nevertheless, despite the potential for significant benefit limitations to biventricular pacing remain. As many as a third of patients treated with conventional CRT do not derive clinical or echocardiographic benefit, and some worsen after resynchronization. Nonresponders include patients with a normal QRS duration and those with right bundle branch block. In addition, the absence of suitable cardiac venous anatomy (lateral leads are optimal; apical and anterior lead position are not) or phrenic nerve stimulation may preclude benefit[12].

A 2022 observational study compared conduction system pacing (CSP) using His-bundle pacing (HBP) or left bundle branch area pacing (LBBAP) to biventricular pacing. Improvement in left ventricular ejection fraction occurred in both groups but was significantly better among CSP recipients. In addition, the primary outcome of death or heart failure hospitalization was significantly lower in the CSP group[13]. HBP has limitations including high pacing thresholds, lead dislodgement, and is less efficacious in the presence of distal conduction system disease compared to left bundle branch pacing (see below).

Despite all the advances made in cardiac pacing, lead related issues remain the "Achilles heel" of cardiac pacing. With the exception of infection, there is a greater incidence of lead related complications compared with issues related to pulse generators. These issues include early dislodgement and loss of capture, fracture of the electrical conduction component leading to high impedance and failure to capture, and deterioration of the lead insulation which frequently presents as "noise" on the lead, oversensing, and absence of pacemaker output. Less frequently, insulation issues may allow a current leak and loss of capture. Deterioration of the lead insulation may allow a current leak and loss of capture, but this more frequently presents as noise on the lead, oversensing, and absence of pacemaker output. While the acute inflammatory reaction associated with lead maturation may result in loss of capture or undersensing, this is usually remediable with programming adjustment. Chronic inflammation (at times due to an underlying

primary cardiomyopathic process) may result in loss of capture. Other problems include infection/endocarditis, venous thrombosis and emboli, and tricuspid regurgitation[14].

In 2016, Ohlow et al. reported a 3.4% incidence of inadvertent lead placement into the left heart, including the cardiac veins[15]. Inadvertent endocardial left ventricular (LV) lead placement creates a nidus for thrombus formation and possible embolization. Treatment of LV lead misplacement discovered late after implantation includes lead removal or chronic anticoagulation with warfarin to prevent thromboemboli. Although LV lead extraction was first described in 1991[16], procedural safety remains uncertain. Because the use of dabigatran in patients with mechanical heart valves was associated with increased rates of thromboembolic and bleeding complications compared with warfarin, substituting a direct oral anticoagulant for warfarin in the setting of malpositioned left ventricular leads is not recommended[17].

References

1. Hyman AS. Resuscitation of the Stopped Heart by Intracardial Therapy: II. Experimental use of an Artificial Pacemaker. Arch Intern Med (Chic). 1932;50(2):283–305. doi:10.1001/archinte.1932.00150150115012
2. Furman S. Early history of cardiac pacing and defibrillation. Indian Pacing Electrophysiol J. 2002 Jan 1;2(1):2-3. PMID: 17006571; PMCID: PMC1569902.
3. Madias C, Trohman RG. Cardiac resynchronization therapy: the state of the art. Expert Rev Cardiovasc Ther. 2014 May;12(5):573-87. doi: 10.1586/14779072.2014.909284. PMID: 24749925.
4. Bigelow WG, Callaghan JC, Hopps JA. General hypothermia for experimental intracardiac surgery; the use of electrophrenic respirations, an artificial pacemaker for cardiac standstill and radio-frequency rewarming in general hypothermia. Ann. Surg. 1950;132:531–539.
5. Zoll PM. Resuscitation of the heart in ventricular standstill by external electric stimulation. N Engl J Med. 1952;247:768–771.
6. Lemberg L, Castellanos A Jr, Berkovits BV. Pacemaking on Demand in AV Block. JAMA. 1965 Jan 4;191:12-4. doi: 10.1001/jama.1965.03080010018003. PMID: 14222963.
7. Sutton RFJ, Linde C, Benditt DG. History of electrical therapy for the heart. Eur Heart J Suppl 2007;9(Suppl I):I3-I10).
8. Cazeau S, Ritter P, Bakdach S, Lazarus A, Limousin M, Henao L, Mundler O, Daubert JC, Mugica J. Four chamber pacing in dilated cardiomyopathy. Pacing Clin Electrophysiol. 1994 Nov;17(11 Pt 2):1974-9. doi: 10.1111/j.1540-8159.1994.tb03783.x. PMID: 7845801.
9. Daubert JC, Ritter P, Le Breton H, Gras D, Leclercq C, Lazarus A, Mugica J, Mabo P, Cazeau S. Permanent left ventricular pacing with transvenous leads inserted into the coronary veins. Pacing Clin Electrophysiol. 1998 Jan;21(1 Pt 2):239-45. doi: 10.1111/j.1540-8159.1998.tb01096.x. PMID: 9474680.
10. Nichols M. European Perspectives. Spotlight: Angelo Auricchio, MD, PhD, FESC. Circulation 2009;120:f85-90.
11. Auricchio A, Klein H, Tockman B, et al. Transvenous biventricular pacing for heart failure: can the obstacles be overcome? Am J Cardiol 1999;83:136D-42D.
12. Sharma PS, Vijayaraman P. His Bundle Pacing Or Biventricular Pacing For Cardiac Resynchronization Therapy In Heart Failure: Discovering New Methods For An Old Problem. J Atr Fibrillation. 2016 Dec 31;9(4):1501. doi: 10.4022/jafib.1501. PMID: 29250259; PMCID: PMC5673318.
13. Vijayaraman P, Zalavadia D, Haseeb A, Dye C, Madan N, Skeete JR, Vipparthy SC, Young W, Ravi V, Rajakumar C, Pokharel P, Larsen T, Huang HD, Storm RH, Oren JW, Batul SA, Trohman RG, Subzposh FA, Sharma PS. Clinical outcomes of conduction system pacing compared to biventricular pacing in patients requiring cardiac resynchronization therapy. Heart Rhythm. 2022 Aug;19(8):1263-1271. doi: 10.1016/j.hrthm.2022.04.023. Epub 2022 Apr 29. PMID: 35500791.
14. Link, MS. Pacing system malfunction: Evaluation and management. Available at: https://www.uptodate.com/contents/pacing-system-malfunction-evaluation-and-management?search=pacing%20lead%20problems&source=search_result&selectedTitle=3%7E150&usage_type=default&display_rank=3. Last accessed 7/5/24.
15. Ohlow MA, Roos M, Lauer B, Von Korn H, Geller JC. Incidence, predictors, and outcome of inadvertent malposition of transvenous pacing or defibrillation lead in the left heart. Europace 2016; 18:1049–1054.
16. Trohman RG, Wilkoff BL, Byrne T, Cook S. Successful percutaneous extraction of a chronic left ventricular pacing lead. Pacing Clin Electrophysiol. 1991 Oct;14(10):1448-51. doi: 10.1111/j.1540-8159.1991.tb04063.x. PMID: 1721124.

17. Trohman RG, Sharma PS. Detecting and managing device leads inadvertently placed in the left ventricle. Cleve Clin J Med. 2018 Jan;85(1):69-75. doi: 10.3949/ccjm.85a.17012. PMID: 29328900.
